# Case Report: Trastuzumab deruxtecan combined with fulvestrant in a patient with HR+/HER2-low metastatic breast cancer

**DOI:** 10.3389/fonc.2026.1957375

**Published:** 2026-09-14

**Authors:** Jiaying Chai, Xinyi Zheng, Longyu Hu, Meiwen Ling, Jingjing Yang, Nana Yu, Chen Gan, Yang Sui, Ling Cheng, Huaidong Cheng

**Affiliations:** 1 Department of Oncology, Shenzhen Hospital, Southern Medical University, Shenzhen, China; 2 Shenzhen School of Clinical Medicine, Southern Medical University, Shenzhen, China; 3 Department of Traditional Chinese Medicine, Shenzhen Pingshan People’s Hospital, Shenzhen, China; 4 Department of Traditional Chinese Medicine, Shenzhen Pingshan Hospital, Southern Medical University, Shenzhen, China

**Keywords:** antibody-drug conjugate, fulvestrant, HER2-low, metastatic breast cancer, trastuzumab deruxtecan

## Abstract

Breast cancer is the most common malignancy among women worldwide, and hormone receptor-positive (HR+)/human epidermal growth factor receptor 2 (HER2)-low breast cancer poses significant challenges once resistance to endocrine therapy and CDK4/6 inhibitors develops. We present a 45-year-old female patient with HR+/HER2-low metastatic breast cancer who progressed on standard-of-care treatments. The combination of trastuzumab deruxtecan and fulvestrant demonstrated significant clinical efficacy and favorable tolerability, achieving a progression-free survival of 26 months with no grade 3 or higher adverse events observed. This case suggests that this combination may represent a promising therapeutic strategy for patients with advanced, treatment-refractory HR+/HER2-low breast cancer, offering a useful reference for clinical practice.

## 1 Introduction

Breast cancer (BC) is the most prevalent malignancy among women globally and the leading cause of cancer-related mortality (1). Despite significant improvements in the long-term prognosis of early-stage BC due to widespread screening and the advancement of multidisciplinary integrated treatments, approximately 20%–30% of patients eventually develop distant metastases (2). Based on the expression status of the estrogen receptor (ER), progesterone receptor (PR), human epidermal growth factor receptor 2 (HER2), and the proliferation index Ki-67, BC is categorized into four primary molecular subtypes: Luminal A, Luminal B, HER2-positive, and triple-negative (3). HER2-low BC (defined as HER2 1+ or 2+ with negative *in situ* hybridization (ISH)) is a clinical subgroup traditionally classified as HER2-negative. Approximately 60% of patients with HER2-negative BC exhibit HER2-low expression, which has now emerged as a critical clinical therapeutic target (4). Currently, the first-line standard of care for advanced disease in this population is endocrine therapy (ET) in combination with a cyclin-dependent kinase 4/6 (CDK4/6) inhibitor (5). Nevertheless, the development of resistance following first-line therapy remains a primary challenge in clinical management.

Antibody-drug conjugates (ADCs) represent a novel class of targeted antineoplastic agents composed of a monoclonal antibody, a chemical linker, and a cytotoxic payload (6). By integrating the precision of antibody-mediated targeting with the potent systemic toxicity of cytotoxic agents, ADCs have demonstrated substantial clinical value within the oncology landscape. In BC treatment, ADCs such as trastuzumab deruxtecan (T-DXd) have delivered substantial survival benefits for patients with advanced disease (7). Currently, a vast array of ADCs remain under development or in clinical trials, underscoring the clinical imperative to explore and optimize their therapeutic strategies.

We present a 45-year-old female patient with hormone receptor-positive (HR+), HER2-low metastatic breast cancer (MBC). Following the failure of multiple lines of therapy, the patient was initiated on a combination regimen of T-DXd and fulvestrant, achieving a marked clinical response and a favorable safety profile. This case report was prepared following the CARE Guidelines.

## 2 Case presentation

### 2.1 Initial diagnosis and treatment

The patient is a 45-year-old female with no significant medical history or family history of malignancy. She presented in January 2021 due to a detected right breast mass. Breast magnetic resonance imaging (MRI) revealed multiple abnormal signals in the right breast, along with enlarged lymph nodes in the right axilla and right subclavian region. Pathology results from right breast lump biopsy confirmed invasive breast carcinoma. Immunohistochemistry (IHC) showed: ER: approximately 60% (2+), PR: approximately 60% (2+), AR: approximately 60% (2+), HER2 (1+), Ki-67: approximately 40% in hot spots (+). Right axillary and subclavian lymph node biopsies were consistent with metastatic adenocarcinoma. Further staging investigations, including head/neck/thorax/abdomen computed tomography (CT) and bone scan, revealed no evidence of metastasis. Pathological staging: cT1N3M0 IIIC; Luminal subtype: Type B. Between January and March 2021, the patient received four cycles of neoadjuvant chemotherapy with nab-paclitaxel, epirubicin, and cyclophosphamide (NabP-EC), which resulted in marked tumor shrinkage on subsequent imaging. In March 2021, the patient underwent modified radical mastectomy of the right breast with concurrent axillary lymph node dissection. Postoperative pathology revealed invasive ductal carcinoma, histological grade II, with vascular invasion. No neoplastic tissue was identified in the fibrofatty tissue. Postoperative immunohistochemistry results: Cancer cells showed ER: (+>80%, moderate/strong), PR: (+30%, moderate), HER2: (+1), CK5/6 (–): Ki-67: (+<10%). Axillary lymph nodes (32/36). Postoperative pathological staging: ypT1N3M0 Stage IIIC. Following surgery, the patient received two additional cycles of NabP-EC and postoperative radiotherapy (50 Gy/25 fractions), with concurrent tamoxifen and goserelin as ET. No evidence of metastasis was observed on regular follow-up imaging.

### 2.2 Brain metastasis and related treatments

In November 2021, follow-up contrast-enhanced brain MRI revealed an isolated metastatic lesion in the right frontal lobe. The patient underwent neuronavigation-guided right frontal craniotomy for resection of a frontal lobe mass. Postoperative pathology confirmed MBC. Following surgery, the patient received treatment with the combination regimen of anastrozole, palbociclib and goserelin. Regular follow-up examinations during this period did not reveal any new lesions. In August 2022, follow-up contrast-enhanced brain MRI demonstrated multiple abnormally enhanced lesions adjacent to the falx cerebri in the right frontal lobe, suggestive of metastasis. She was subsequently treated with fractionated stereotactic radiotherapy (60Gy/30 fractions) concurrent with two cycles of temozolomide, achieving a partial response. In May 2023, the patient experienced recurrent seizures. Brain MRI revealed abnormal enhancement foci in the paracerebellar regions of both frontal lobes and beneath the medial table of the right frontal bone (**Figure 1**), raising suspicion of metastasis. The treatment regimen was adjusted to the combination of exemestane, abemaciclib and goserelin. Three months later, the patient underwent a second resection of the right frontal lobe metastasis. Postoperative pathology confirmed BC brain metastasis. IHC results showed: ER (+60%, moderate), PR (+10%, weak), HER2 (2+), ISH (-), Ki-67 (+70%). Postoperatively, treatment was initiated with the combination regimen of Abemaciclib, Exemestane, Pyrotinib and Goserelin.

**Figure 1:**
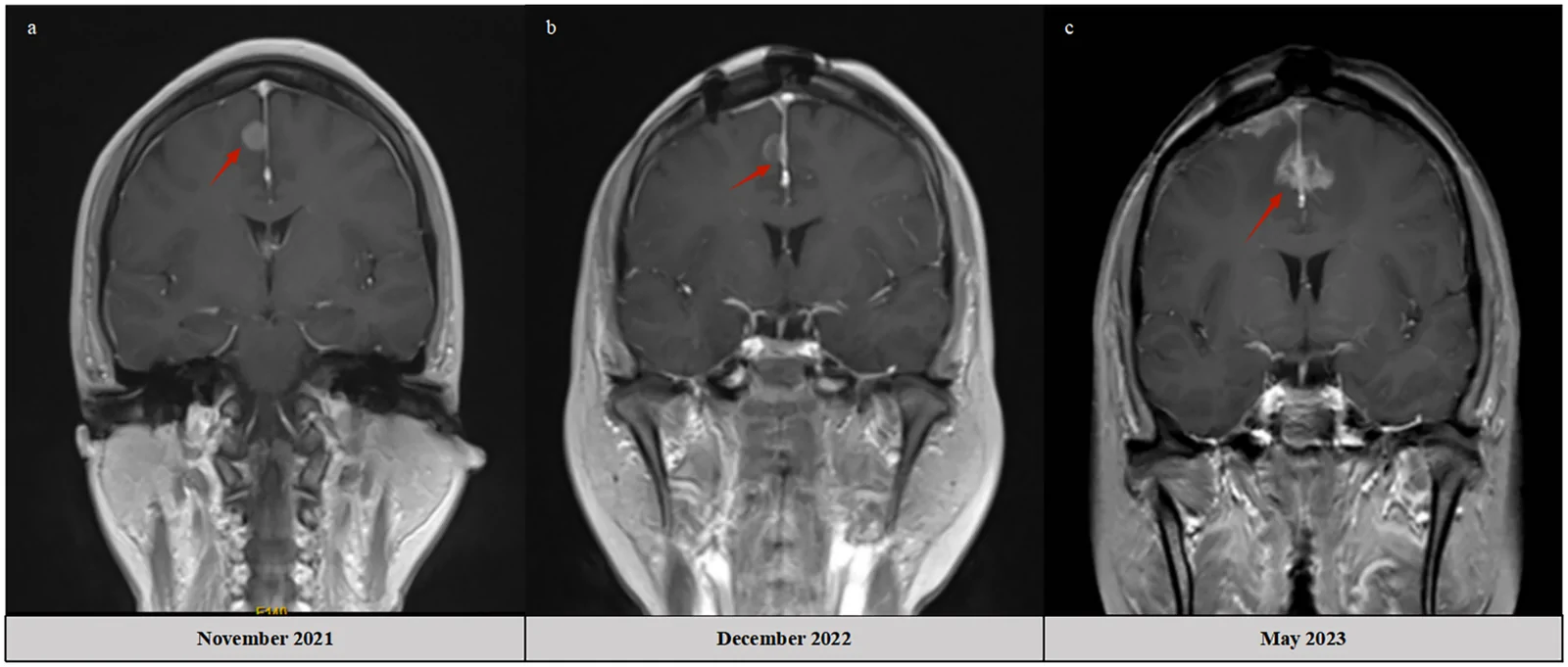
MRI evaluation of brain metastases. **(a)** In November 2021, the patient’s brain MRI revealed an isolated metastatic lesion in the right frontal lobe. **(b)** The patient underwent stereotactic body radiation therapy for an intracranial lesion, and follow-up evaluation showed partial response. **(c)** In May 2023, the patient experienced simple partial seizures. Brain MRI findings suggested disease progression.

### 2.3 Widespread metastasis and combined therapy

In January 2024, follow-up brain MRI demonstrated an expansion of the patchy cerebral edema in the bilateral frontal lobes. Multiple metastatic lesions were identified in the perisylvian region and right frontal-parietal meninges. Multiple patchy hyperenhancing foci in the skull were noted, suggestive of metastatic tumors. Chest and abdominal CT scans revealed multiple liver metastases, with the largest measuring approximately 33 mm × 27 mm. Abnormal bone density was noted in the sternum, multiple thoracolumbar vertebrae, sacrum, sacral promontory, left ilium, left ribs (2nd, 3rd, 5th, 6th), and right 11th posterior rib, consistent with metastatic lesions. Comprehensive assessment indicated overall disease progression, treatment with abemaciclib, exemestane and pyrotinib was discontinued. In January 2024, the patient switched to combined therapy with T-DXd (5.4 mg/kg every 3 weeks) and fulvestrant (500 mg every 4 weeks), concurrently receiving denosumab for bone protection. Regular imaging and laboratory tests were conducted during treatment to evaluate efficacy. Extracranial and intracranial responses were assessed per RECIST 1.1 and RANO-BM, respectively. After completing 4 cycles, both brain and liver metastases achieved partial responses. Following 6 months of sustained partial response, the dosing schedule was adjusted to an extended interval of 5.4 mg/kg every 4 weeks. At a follow-up in April 2025, liver metastases achieved a complete response per RECIST 1.1, while brain metastases maintained a partial response per RANO-BM. Unfortunately, subsequent imaging in March 2026 confirmed progressive disease. A brain MRI revealed an increase in both the size and number of intracranial lesions(**Figure 2**), and an abdominal CT demonstrated recurrent liver metastases alongside newly developed adrenal metastases (**Figure 3**).

**Figure 2:**
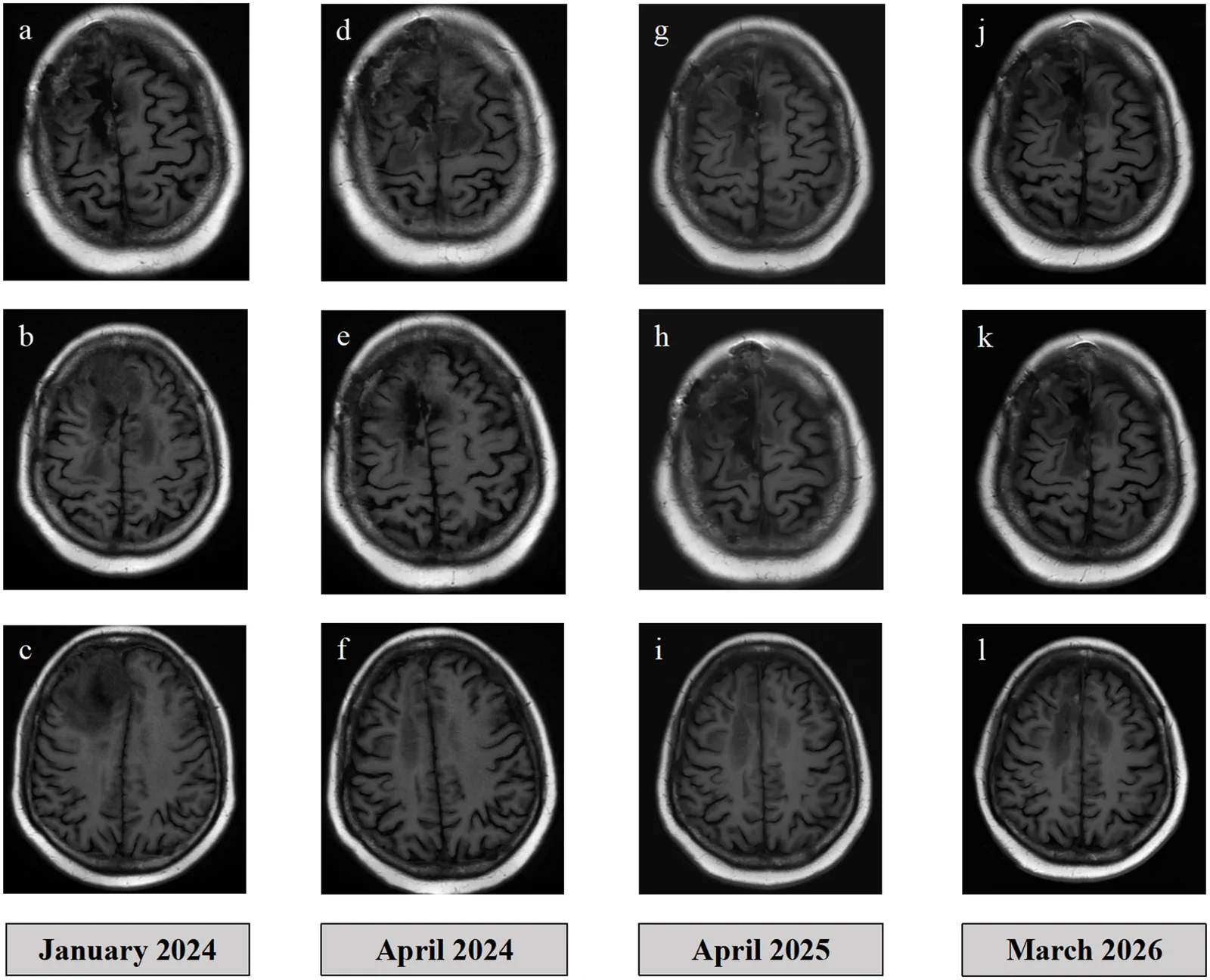
MRI response evaluation in brain metastases during Trastuzumab Deruxtecan and Fulvestrant combination therapy. **(a–c)** MRI evaluation in January 2024 provided a baseline assessment of the patient’s brain metastases. **(d-f)**: MRI evaluation in April 2024 showed that after four cycles of treatment, the patient achieved a partial response in brain metastases. **(g–i)** MRI evaluation in April 2025 demonstrated sustained disease control. **(j–l)** MRI evaluation in March 2026 revealed disease progression.

**Figure 3:**
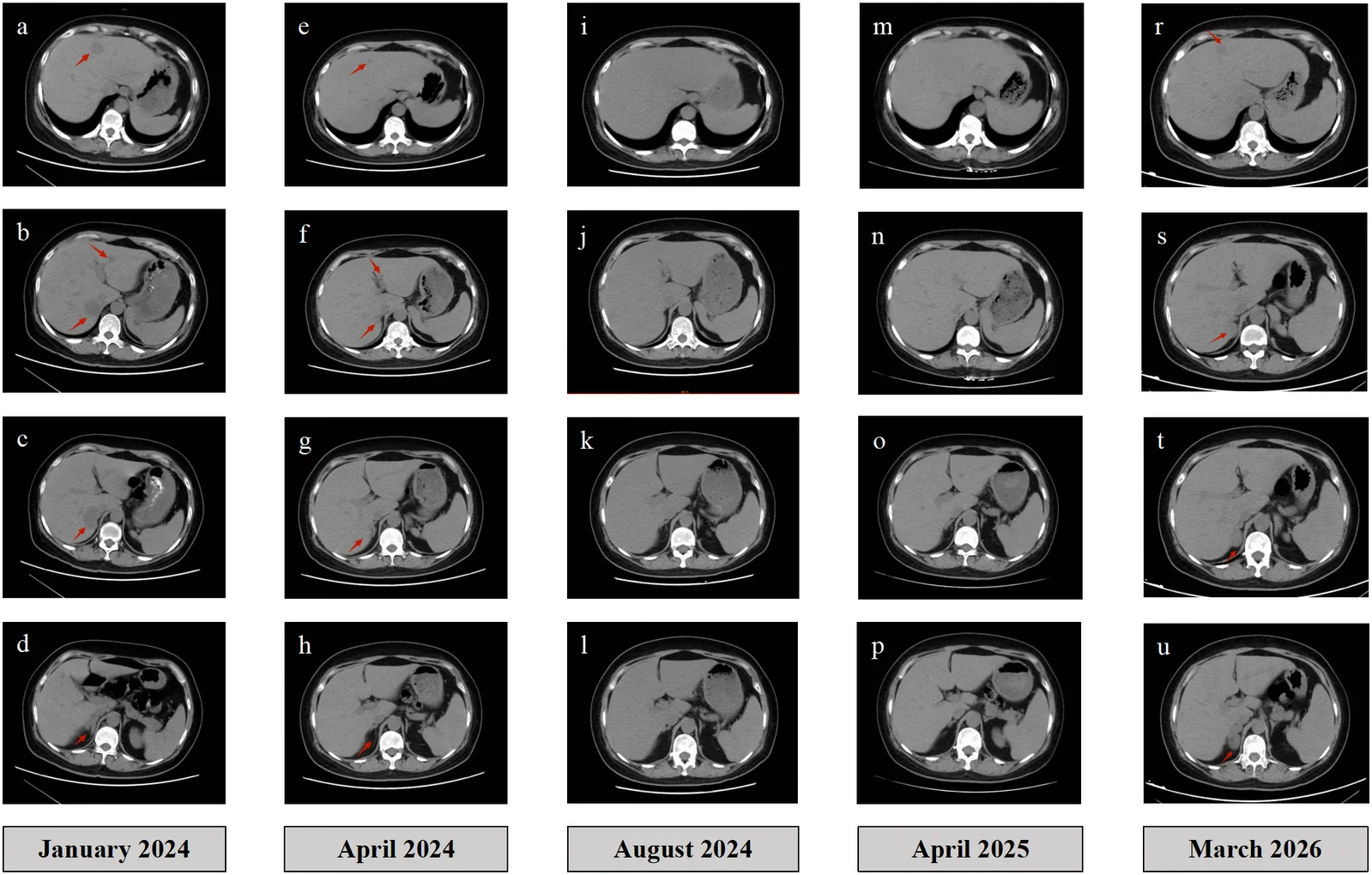
CT response evaluation in liver metastasis during Trastuzumab Deruxtecan and Fulvestrant combination therapy. **(a–d)** CT in January 2024 provided a baseline assessment of the patient’s liver metastases. **(e–h)** CT evaluation in April 2024 showed that after four cycles of treatment, the patient achieved a partial response in liver metastases. **(i–l)** CT evaluation in August 2024 showed that the patient achieved a complete response in liver metastatic lesions. **(m–p)** CT evaluation in April 2025 demonstrated sustained complete response. **(r–u)** CT evaluation in March 2026 revealed disease progression, demonstrating recurrent liver metastases alongside newly developed adrenal metastases.

The complete treatment timeline is summarized in **Figure 4**. The patient exhibited favorable tolerance to the combination therapy of T-DXd and fulvestrant. According to the Common Terminology Criteria for Adverse Events (CTCAE, version 5.0), no grade 3 or higher adverse events (AEs) were observed. Grade 2 neutropenia and thrombocytopenia were effectively managed, and no other significant complications occurred. Throughout the course of the combination therapy, the patient’s general condition remained stable with an Eastern Cooperative Oncology Group Performance Status (ECOG PS) score of 1. Neurological symptoms were well-controlled, enabling the patient to perform activities of daily living independently and resulting in a marked improvement in quality of life.

**Figure 4:**
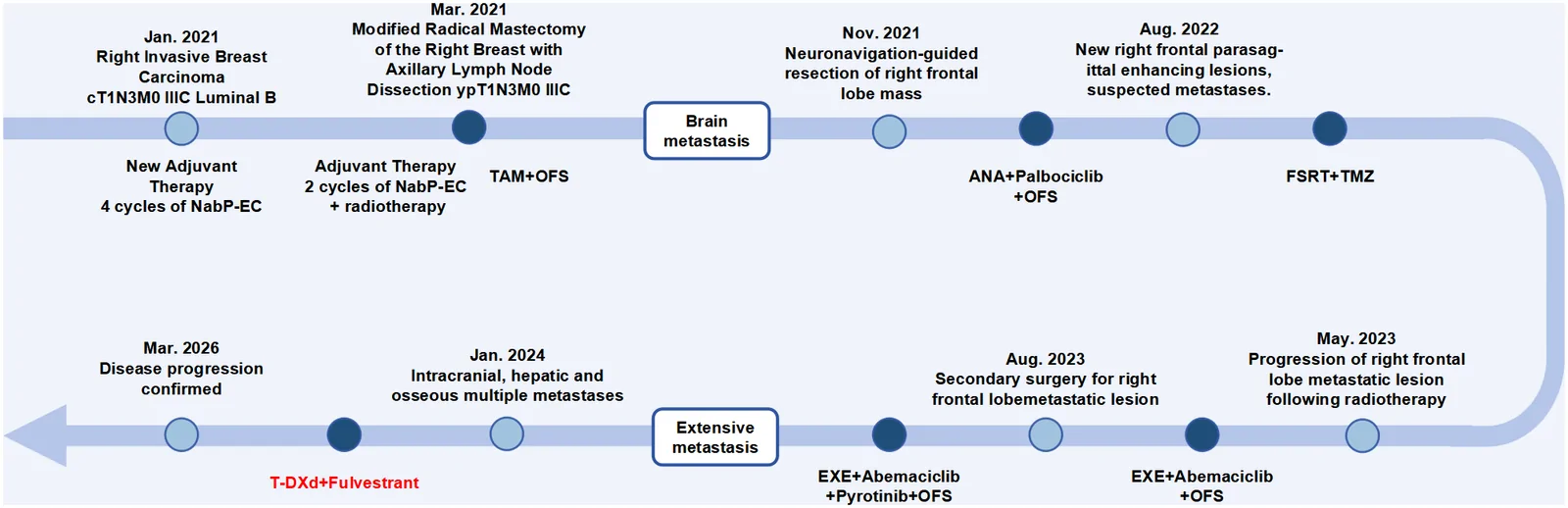
The treatment timeline for this patient. NabP-EC, nab-Paclitaxel + Epirubicin + Cyclophosphamide; TAM, Tamoxifen; OFS, Ovarian Function Suppression; ANA, Anastrozole; EXE, Exemestane; TMZ, Temozolomide; T-Dxd, Trastuzumab Deruxtecan; FSRT, Fractionated Stereotactic Radiotherapy.

### 2.4 Patient perspective

“Initially, I was anxious about starting a new combination therapy after my disease progressed. However, over the past two years of treatment, my condition has remained stable, and most notably, my seizures have been effectively controlled. My quality of life has improved, allowing me to continue my daily activities. Although I experienced some side effects, they were effectively managed by my medical team. I am deeply grateful for the care and support I have received.

## 3 Discussion

Optimizing treatment sequencing after ET and CDK4/6 inhibitor progression remains a major challenge in HR+/HER2-low MBC. We report a 45-year-old female with extensive brain, liver, and bone metastases refractory to multiple standard therapies, including neoadjuvant chemotherapy, surgery, radiotherapy, and ET regimens. In this patient, a combination of T-DXd and fulvestrant yielded a profound, durable response. This single-case observation suggests a potential benefit of an ADC plus ET dual-blockade strategy in addressing tumor spatial heterogeneity and cross-pathway resistance, supporting further exploration of this approach in later-line HR+/HER2-low MBC.

### 3.1 Contextualization within existing trial evidence

HR+/HER2-negative BC accounts for approximately 70% of all breast malignancies. Although ET combined with CDK4/6 inhibitors represents the current standard first-line treatment, therapeutic options following disease progression remain limited (8). In recent years, ADCs have emerged as a pivotal modality in precision oncology, owing to their dual advantages of precise targeting and potent cytotoxicity. DESTINY-Breast04, the landmark Phase III clinical trial establishing the efficacy of T-DXd in the HER2-low population, demonstrated that T-DXd yielded statistically significant improvements in both progression-free survival (PFS) and overall survival (OS) compared to standard chemotherapy (9).

Furthermore, T-DXd has shown substantial activity against central nervous system (CNS) metastases in BC. In the DESTINY-Breast04 trial, a subgroup analysis of patients with baseline brain metastases revealed a 12-month PFS rate of 61.6%, a CNS PFS rate of 58.9%, and a CNS objective response rate (ORR) of 25% (including a 16.7% intracranial complete response) (10, 11). Additionally, the DEBBRAH trial confirmed T-DXd’s efficacy in HER2+ or HER2-low BC with leptomeningeal carcinomatosis, yielding a median PFS of 8.9 months and a median OS of 13.3 months, further corroborating its value in managing intracranial disease (12). T-DXd’s superior efficacy is driven by its unique pharmacological properties. As a HER2-targeted ADC with a high drug-to-antibody ratio (DAR ≈ 8), it delivers a potent topoisomerase I inhibitor payload into HER2-low tumor cells (13). Crucially, its membrane-permeable payload exerts a robust “bystander effect,” eradicating adjacent cells regardless of HER2 expression and overcoming intratumoral heterogeneity (14). Additionally, T-DXd effectively penetrates the blood-brain barrier (BBB) to achieve therapeutic concentrations within intracranial lesions (13).

Based on the positive results of the DESTINY-Breast06 trial, the National Medical Products Administration (NMPA) approved T-DXd for patients with HR+/HER2-low (IHC 1+ or IHC 2+/ISH−) or HER2-ultralow MBC who have progressed on one or more lines of ET. To further optimize outcomes, T-DXd combinations are under active investigation. Fulvestrant, a selective estrogen receptor downregulator (SERD), has proven efficacy in ET-resistant HR+ MBC (15, 16). Previous studies highlight a bidirectional crosstalk between the ER and HER2 pathways: ET pressure can upregulate HER2 expression (17–19), while anti-HER2 agents can, in turn, reverse endocrine resistance (20). Therefore, maintaining endocrine blockade alongside ADC therapy represents a promising strategy to overcome cross-pathway resistance. In the Phase Ib DESTINY-Breast08 trial, T-DXd plus fulvestrant demonstrated a 40% confirmed objective response rate (cORR) in HER2-low MBC, with partial response as the best overall response, however, this cohort also reported a 100% treatment-related adverse events (TRAEs) rate, with 55% being grade ≥3 (21).

### 3.2 Case interpretation and limitations

In our case, following the combination therapy, the patient’s multi-site metastases were effectively controlled with a PFS of 26 months, further supporting the known “bystander effect” and CNS penetrability of T-DXd. Notably, our patient achieved a complete hepatic response with this specific combination. However, as a single-case observation, it cannot be fully compared with existing clinical trial data, nor is it sufficient to confirm definitive synergy. Nevertheless, this result highlights a favorable clinical outcome in a real-world setting, suggesting a potential benefit of this dual-blockade strategy and supporting its continued clinical investigation.

Although our patient exhibited primary endocrine resistance, relapsing within six months of adjuvant ET and progressing on an ET/CDK4/6 inhibitor combination, her resected brain metastasis maintained 60% ER positivity and remained targetable by fulvestrant. Furthermore, the patient’s HER2 status differed between the primary tumor (IHC 1+) and the subsequent brain metastasis (IHC 2+, ISH-negative) following multi-line ET. We hypothesize that this phenotypic change might have contributed to the favorable therapeutic response to the combined regimen. However, we must explicitly acknowledge that the clinical significance of this change remains uncertain, as it is an inference retroactively applied to an IHC change between two non-contemporaneous biopsies from different anatomic sites. Such discrepancies can be confounded by inherent tumor spatial heterogeneity, temporal clonal evolution under multi-line treatments, and pre-analytical variations in tissue processing and staining.

Regarding safety, balancing efficacy and tolerability is critical in heavily pretreated MBC. Our patient achieved excellent tolerability with a tailored regimen. After eight cycles yielded a hepatic complete response and a sustained intracranial partial response, her T-DXd interval was extended to every four weeks (Q4W) without dose reduction. Under this regimen, TRAEs were limited to grade 2 neutropenia and thrombocytopenia, with no interstitial lung disease (ILD) or grade ≥3 events. Given her durable disease control, transitioning to a Q4W interval appeared to be well-tolerated and may help balance long-term efficacy and safety, warranting further evaluation as a potential maintenance strategy.

Despite the favorable clinical outcomes, this report presents inherent limitations. First, the single-case design restricts the generalizability of the findings. Second, the concurrent use of T-DXd and fulvestrant precludes delineating their individual therapeutic contributions. Furthermore, the absence of longitudinal molecular evidence, including repeat biopsies, ctDNA monitoring, and cerebrospinal fluid pharmacodynamic assays, prevents definitive validation of the proposed mechanisms.

## 4 Conclusion

In conclusion, this case suggests a potential clinical benefit and manageable tolerability of the T-DXd and fulvestrant combination in heavily pretreated HR+/HER2-low MBC. In the future, large-scale, targeted clinical trials should be conducted to validate the efficacy and safety of this combination therapy. Furthermore, incorporating longitudinal biomarker monitoring, such as serial biopsies or ctDNA analysis, into future studies will be crucial to elucidate the underlying biological mechanisms and guide more individualized therapeutic decisions.

## Data Availability

The original contributions presented in the study are included in the article/supplementary material, further inquiries can be directed to the corresponding author/s.
